# Supplementary material for: A Comparative Study of Clinical and Molecular Features of Microsatellite Stable Colorectal Cancer With and Without Liver Metastases
Source: Cancers (Basel). 2025 Nov 17;17(22):3677. doi: 10.3390/cancers17223677 (PMC12650924; doi:10.3390/cancers17223677)

**Table S1: Results of Cox regression analysis, multivariate Cox regression analysis, and Kaplan-Meier survival analysis assessing the impact of age, CRC location, and molecular alterations on time from first-line to second-line treatment in patients with advanced CRC with liver metastasis and non-liver metastasis**					
**Liver metastasis cohort**					
**Variable**	**Cox regression analysis**		**Multivariate Cox regression analysis**		**Kaplan-Meier survival analysis**
	**HR**	**p-value**	**HR**	**p-value**	**p-value**
**Age at analysis**	1.00	0.5	N/A	N/A	N/A
**Site of primary tumor (rectum/colon)**	1.29	0.3	N/A	N/A	0.28
**KRAS**	1.53	0.012	1.56	0.012	0.012
**NRAS**	1.04	>0.9	0.97	>0.9	0.92
**BRAF**	1.11	0.8	1.38	0.4	0.79
**TP53**	0.96	0.8	1.05	0.8	0.83
**BRCA2**	1.08	0.9	0.99	>0.9	0.85
**PIK3CA**	1.22	0.4	1.13	0.6	0.42
**TMB (categorical)**	1.23	0.2	N/A	N/A	0.22
**TMB (continuous)**	1.02	0.2	N/A	N/A	N/A
**Non-liver metastasis cohort**					
**Variable**	**Cox regression analysis**		**Multivariate Cox regression analysis**		**Kaplan-Meier survival analysis**
	**HR**	**p-value**	**HR**	**p-value**	**p-value**

**Age at analysis**	1.00	0.9	N/A	N/A	N/A
**Site of primary tumor (rectum/colon)**	0.81	0.5	N/A	N/A	0.45
**KRAS**	1.32	0.3	1.54	0.2	0.3
**NRAS**	0.94	>0.9	1.28	0.7	0.9
**BRAF**	1.28	0.6	1.64	0.3	0.56
**TP53**	0.73	0.3	0.73	0.3	0.25
**BRCA2**	N/A	N/A	2.18	0.3	N/A
**PIK3CA**	1.13	0.7	0.85	0.7	0.74
**TMB (categorical)**	0.75	0.3	N/A	N/A	0.28
**TMB (continuous)**	N/A	N/A	N/A	N/A	N/A

**Table S2: First-line systemic treatments of patients with advanced CRC**			
**First-line treatment**	**Liver metastasis** (N=148)	**Non-liver metastasis** (N=61)	**Overall** (N=209)
**FOLFOX**	80 (54%)	38 (62%)	118 (56%)
**FOLFOX and bevacizumab**	25 (17%)	4 (6.6%)	29 (14%)
**XELOX**	6 (4.1%)	6 (9.8%)	12 (5.7%)
**FOLFOX and panitumumab**	8 (5.4%)	0 (0%)	8 (3.8%)
**FOLFIRI**	8 (5.4%)	0 (0%)	8 (3.8%)
**FOLFOXIRI**	6 (4.1%)	1 (1.6%)	7 (3.3%)
**FOLFIRI and bevacizumab**	2 (1.4%)	4 (6.6%)	6 (2.9%)
**FOLFIRINOX**	4 (2.7%)	0 (0%)	4 (1.9%)
**Capecitabine**	2 (1.4%)	2 (3.3%)	4 (1.9%)
**FOLFOX and cetuximab**	3 (2.0%)	0 (0%)	3 (1.4%)
**FOLFIRI and panitumumab**	1 (0.7%)	2 (3.3%)	3 (1.4%)
**5-FU**	0 (0%)	1 (1.6%)	1 (0.5%)
**5-FU/leucovorin plus bevacizumab**	1 (0.7%)	0 (0%)	1 (0.5%)
**Bevacizumab**	0 (0%)	1 (1.6%)	1 (0.5%)

**Capecitabine and bevacizumab**	0 (0%)	1 (1.6%)	1 (0.5%)
**CAPOX**	0 (0%)	1 (1.6%)	1 (0.5%)
**FOLFIRI + Vectibix**	1 (0.7%)	0 (0%)	1 (0.5%)
**Pembrolizumab**	1 (0.7%)	0 (0%)	1 (0.5%)

Figure S1: Median Overall Survival with BRAF status among patients with liver metastases.

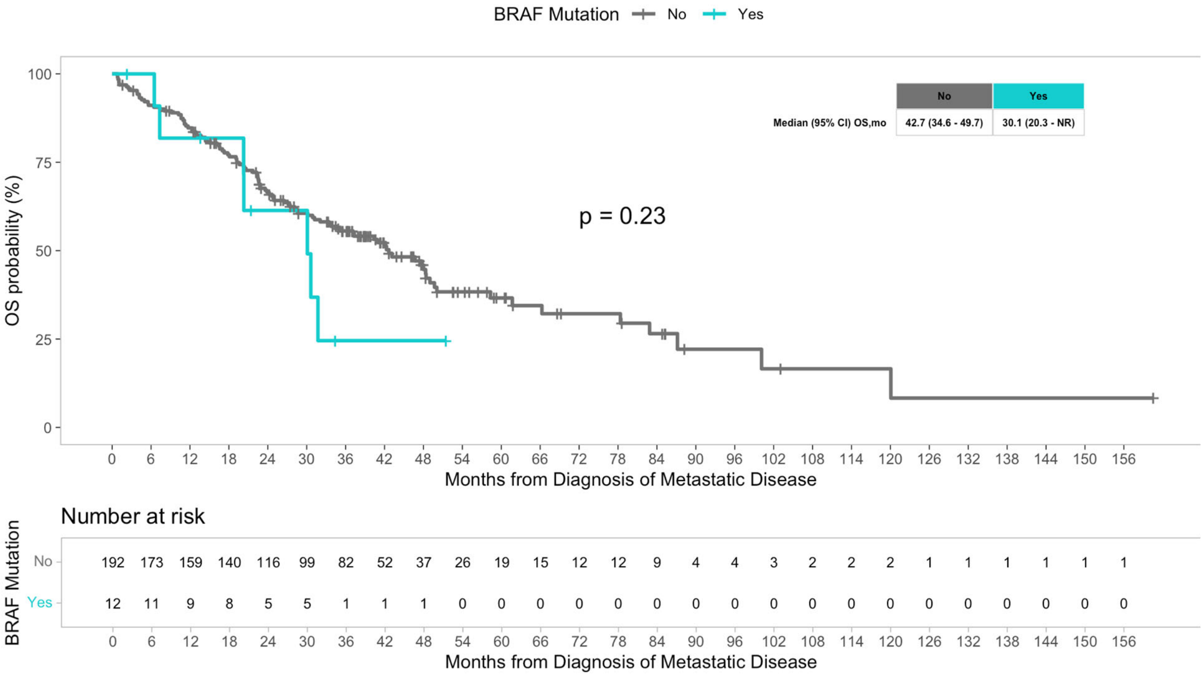

Figure S2: Median time from first-line to second-line treatment in patients with advanced CRC with liver metastasis with KRAS mutations versus without KRAS mutations

Kaplan-Meier Plot of Treatment-free Survival by KRAS Mutation Status in Patients With Liver Metastasis

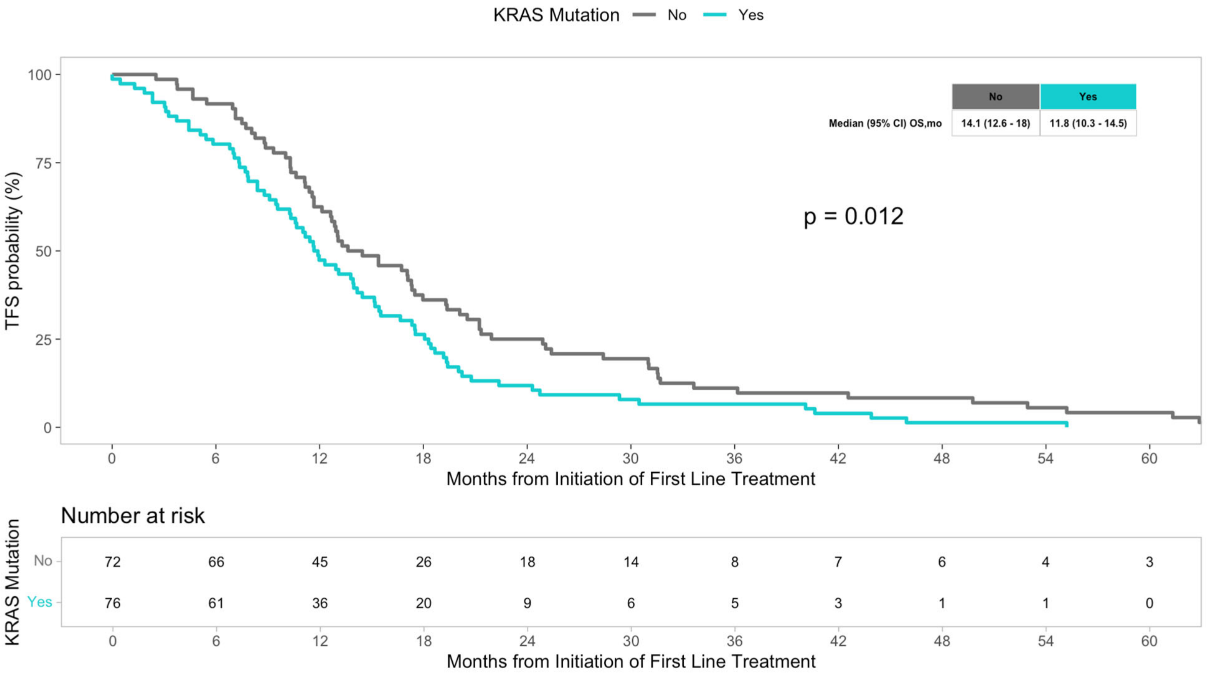

Supplement: Supplementary file 1 [file cancers-17-03677-s001.zip › cancers-3950827-supplementary.pdf]
